# Data science and artificial intelligence in biology, health, and healthcare

**DOI:** 10.1017/cts.2025.28

**Published:** 2025-02-14

**Authors:** Peter L. Elkin, Christopher Lindsell, Julio Facelli, Manisha Desai, Chunhua Weng, Heidi Spratt, Shari Messinger, Lemuel Russell Waitman, JaMor Hairston, Ruth O’Hara, Jareen Meinzen-Derr

**Affiliations:** 1 Department of Biomedical Informatics, Jacobs School of Medicine and Biomedical Sciences, University at Buffalo, Buffalo, NY, USA; 2 Duke University, Durham, NC, USA; 3 Department of Biomedical Informatics, University of Utah, Salt Lake City, UT, USA; 4 Stanford University School of Medicine, Stanford, CA, USA; 5 Department of Biomedical Informatics, Columbia University, New York, NY, USA; 6 Department of Biostatistics and Data Science, University of Texas Medical Branch, Galveston, TX, USA; 7 Department of Public Health Sciences, Division of Biostatistics and Bioinformatics, University of Miami, Coral Gables, FL, USA; 8 Department of Biomedical Informatics, Biostatistics and Medical Epidemiology, Missouri University School of Medicine, Cincinnati, OH, USA; 9 Department of Biomedical Informatics, Emory University School of Medicine, Birmingham, AL, USA; 10 Department of Pediatrics, Cincinnati Children’s Hospital Medical Center, University of Cincinnati College of Medicine, Cincinnati, OH, USA

**Keywords:** Data science, biomedical informatics, biostatistics, training, research

Biomedical science is at an inflection point where classical thinking about how to conduct translational research is undergoing a transformation. Enabled by now-available data sources combined with exponential advances in AI, Statistics, and Computer Science, we can achieve advances that require levels of complexity that we could not have previously thought possible [1]. To investigate modern biomedical questions of significance requires data scientists to serve as partners in data-driven science, as well as continuing their leadership in data science, biomedical informatics, and biostatistics research [2]. The future is hobbled by an insufficient data science workforce cross-trained in the biomedical sciences. As resources shift to centralized and connected repositories of increasingly complex data, the data science and AI community urges immediate action to ensure we draw unbiased conclusions from the vast information available to modern researchers [3]. Specifically,

- In order for science to be data-driven, qualified data scientists should be integrally engaged from the project onset to limit fatal flaws in methodology, analysis, and model development.
- Major data science initiatives should be coordinated to prevent redundancy and inefficiencies. This includes the consistent use of commonly accepted standards for codifying health data.
- It is critical to rapidly grow a qualified workforce of data scientists who can be counted on to exhibit a common core set of competencies, as well as to provide quantitative science training in the biomedical science curricula.
- Artificial Intelligence (AI) in biomedicine is a rapidly evolving field with significant promise and also significant risk. Attention to the elimination of bias, Ethical Legal and Social Issues (ELSI), the selection of data and populations on which to train models and other issues must continue to be addressed.
- As we learn how to safely and effectively train and use AI in healthcare and research applications, it is essential that we establish frameworks for evaluating when and if data science methods and AI are fit for purpose.
- Emphasizing the FAIR principles (Findable, Accessible, Interoperable and Reusable), along with data cleaning, storage, indexing and data exchange and accessibility of data fit for purpose should be emphasized. Publishing high-quality data resources should be rewarded similarly to publications in peer-reviewed journals.
- Congress should increase the NIH budget specifically earmarked for data science research and education, informatics, and biostatistics to address this immediate and critical need. We suggest that good homes for the funding include NCATS, NLM, and then other interested ICs.

1. Set expectations for the early inclusion of data scientists in all clinical biomedical research.

Data scientists provide novel and independent contributions requiring a deep knowledge base and creativity. While team science is lauded, until data scientists are recognized for their pivotal contributions as collaborating scientists on the team, there will continue to be a barrier for data scientists to focus on addressing biomedical questions. Funding agencies can continue to help address this barrier by ensuring that data-centered science includes data scientists in named, recognized leadership roles, such as multi-PI roles, as is being increasingly observed.

1. Coordinate major data science and AI initiatives among federal agencies

There are increasing demands on the biomedical research community to meet the data science needs of multiple federal agencies. When multiple common data models are imposed, it creates competing workstreams for an already overburdened and under-supported health information technology ecosystem and for the data science workforce. Lack of consistency creates inefficiencies, and sharing of noninteroperable data can lead to errors in reporting. Mapping between data standards, data repositories, and common data models creates efficiency but at the cost of information and quality. Deciding on a common set of interoperability standards would be the true path to semantic interoperability for our research and clinical care enterprise. Semantic Interoperability requires formalisms well beyond selecting a common data model.

1. Grow the data science and AI workforce and establish core competencies and subspecialties for data scientists in the fields of biomedical research.

There is a national shortage of data scientists to meet the needs of the future as envisioned. This deficit can be addressed both by creating new training programs and expanding existing programs. As established and emerging data sciences including AI and machine learning evolve, recognition of subspecialty skills when forming scientific teams is critical – biostatisticians, informaticians, and data engineers are not interchangeable. The paradigm for training may also need to evolve: as programs sprint to keep pace with rapidly changing technology it is critical to ensure that the core competencies needed for extracting knowledge from data are addressed alongside the range of technical proficiencies that can be achieved. Doctoral, postdoctoral, and continuing data science training and education programs should consider the sheer breadth of the activities that constitute data science and ensure a common understanding of the general and specialty-specific knowledge base and competencies needed to train a functional data scientist workforce [4]. We would seek to create a generation of life-long learners.

Addressing these challenges will require a major coordinated response from academia, industry, and government agencies. The many existing efforts can be optimized for impact with a clearer recognition and understanding of both core competencies and subspecialty skills, by applying standards for technology stacks and data stores, and by providing a reward and value system for data scientists contributing to biomedical research. Without a coordinated focus on these issues, we expect the gaps in expertise across the research enterprise to widen. However, greater attention to the biomedical data science workforce has the potential to catalyze efficiencies and shorten timelines to have robust answers to biomedical questions bringing new treatments to patients more rapidly. Strong data science is expected to result in not only high-quality reproducible research but also a transformation of how clinical and translational research is performed. Specifically, we can use existing data to help us understand what studies are more likely to show positive results. We can judge likely toxicities ahead of expensive phase III clinical trials, and in so doing limit the number of negative trials. We can improve recruitment to execution of clinical trials using real-world evidence. We can more effectively do postmarket surveillance [5] to find rare but serious side effects and deliver the right treatments to the right patients at the right time.
